# The supply-side effects of cannabis legalization

**DOI:** 10.1186/s42238-022-00148-7

**Published:** 2022-07-22

**Authors:** Joohun Han, John N. Ng’ombe

**Affiliations:** 1 Department of Agricultural Economics, Oklahoma State University, Stillwater, OK 74078 USA; 2 Department of Agribusiness, Applied Economics, and Agriscience Education, North Carolina A&T State University, Greensboro, NC 27411 USA

**Keywords:** Cannabis market, Cannabis legalization, Recreational cannabis, Non-linear programming, Nested CES function, The USA, Oklahoma

## Abstract

**Objective:**

The purpose of this study is to examine how cannabis legalization and corresponding taxation would affect the supply-side of the cannabis market. Specifically, the study considers various scenarios in which Oklahoma legalizes recreational cannabis for adult use and simulates changes in state-level market sales for other legal states and the average grower profits in Oklahoma. We assume that legalizing recreational cannabis in medical-only states would significantly increase the demand quantity in the legalized states and the local government would levy a significant level of tax on recreational cannabis. These assumptions are based on the post-legalization phenomena in other legalized US states.

**Method:**

We simulate outcomes in the cannabis industry under the assumption of representative consumers with constant elasticity of substitution demand behavior and profit-maximizing firms with a Cobb-Douglas profit function. All agents are assumed to take exogenous prices as given. We calibrate the model using state-level sales data from 2020 and explore potential policies in Oklahoma and at the federal level.

**Results:**

We find that, under the scenarios we consider, legalization of recreational cannabis in Oklahoma would lead to a decrease in the quantity of cannabis sold in Oklahoma’s medical cannabis market as well as decreases in the quantity of cannabis sold in other states on average. Furthermore, we find that as the excise tax rate on recreational cannabis in Oklahoma is increased, the demand quantity in recreational cannabis market would decrease while the other markets’ demand quantity would increase on average. As the elasticity of substitution between state-level products increases, the overall demand quantity would increase and the market quantity across states become more sensitive to Oklahoma’s tax policies. This pattern could become starker as the elasticity of substitution between recreational and medical cannabis increases. In terms of profit, heavy taxation and price decrease due to legalization would significantly decrease cannabis producers’ production and profit levels unless the cost reduction strategies complement legalization.

**Conclusion:**

Based on our results, the legalization of recreational cannabis has the potential to generate tax revenue to fund critical government projects and services. However, such legalization would have to be done carefully because heavy excise taxes would decrease the legal cannabis market demand and growers’ profit, which would incentivize producers and consumers to move to the illicit cannabis market. Policymakers would have to compromise between the levels of interstate transportation and taxation to ensure that cannabis suppliers also realize some profit within the cannabis supply chain.

## Introduction

Cannabis is the most commonly used illegal drug in the world (Caulkins et al. 2016), and its legalization is unsurprisingly one of the controversial subjects that has gained wider interest from both policymakers and voters in the USA. The goal of medical and recreational cannabis legalization policies is to increase social welfare by preventing cannabis-related illegal activities and substance abuse (Kamin 2016). Nonetheless, concerns about the adverse effects of cannabis legalization such as deleterious health consequences from substance abuse and increased social costs continue to be raised (Volkow et al. 2014). Hall and Lynskey (2016) list car crash fatalities and injuries and the prevalence of regular cannabis use among young people in the criminal justice system as some of the potentially harmful consequences of cannabis legalization policies in the USA. As such, studies have focused on establishing cannabis use perceptions, effective, and efficient legal designs moving forward (e.g., Caulkins et al. 2019; Davenport 2019; Kilmer 2014 and others). Yet, no strong evidence exists about whether the less-restrictive cannabis policy would lead to positive or negative consequences (Cambron et al. 2017).

Cannabis legalization requires that cannabis is being produced, sold and possessed or used legally under the legalization act of that country or state (Caulkins et al. 2016). This means that for precise policy evaluation, the supply-side change should be accounted. Caulkins et al. (2016) contend that legalization would substitute unlawful market production and distribution with an aboveboard industry, which principally highlights the importance of cannabis supply and demand. Importantly and not coincidentally, one of the arguments advanced for the lack of solid evidence about the effectiveness as well as mixed findings of previous cannabis legalization research is that the supply side is often neglected in both research and policy design (Hunt and Miles 2015). Cambron et al. (2017) and Hunt and Miles (2015) even stress that previous research’s silence on the supply side has contributed to making it difficult for one to distinguish between legal medical markets and illegal recreational markets.

Therefore, it follows that predicting more accurate welfare changes associated with cannabis legalization requires one to evaluate all changes in cannabis supply and demand. To the best of our knowledge and as observed by Cambron et al. (2017), empirical studies that examine the effects of cannabis legalization on the supply side of the cannabis market are scanty. Thus, as an increasing number of jurisdictions worldwide consider various liberalizations of cannabis policy, considerable interest in understanding the impacts of such policy changes on the supply side cannot be understated.^1^ This study examines how cannabis legalization impacts the supply side of the market. Specifically, we examine how the change in the legal status of cannabis from medical-only to medical and recreation affects the cannabis market quantity, profit, and corresponding tax revenue. This way, the study contributes to the body of existing cannabis literature by providing potential cannabis supply-side legalization policy implications to authorities in Oklahoma, other US states, and other countries (e.g., Australia) that have similar cannabis legalization plans.

To examine how legalization affects the supply side of the cannabis market, we employ the Oklahoma cannabis market case. The US state of Oklahoma is an ideal environment to simulate how legalization would affect the supply side of the cannabis market because the state recently entered the cannabis market. Specifically, the cannabis market in Oklahoma is legal, yet restricted to medical usage, and corresponding regulations focus on limiting the supply side rather than the demand side. Therefore, we could speculate that simulation results would indicate supply-side changes due to the changes in the cannabis legal status. Furthermore, results would be less dependent on other externalities such as the historical complexity of the policy’s long implementation period. In sum, our results suggest that potential legalization of recreational cannabis use would generate significant tax revenue in the legalized region while decreasing the other states’ demand quantity in general. In addition, the corresponding excise tax would decrease the newly legalized local market quantity and profits for cannabis growers in the short run.

## Background

A state ballot measure known as Oklahoma State Question (OSQ) 788 was issued on April 11, 2016, and approved on June 26, 2018. It legalized production and consumption of medical cannabis in Oklahoma. With this act, individuals or entities with a license are allowed to cultivate, retail, and transport cannabis. In addition, individuals aged over 18 years old with a physician’s prescription are able to possess cannabis in 3 oz daily quota (notice that it only limits how much you can hold, not how much you can use). Besides, with the signing of Bill HB 2612 in March 2019, Oklahoma citizens are allowed to possess up to 8 oz of marijuana at residence. Subsequently, medical cannabis patient numbers also increased from 220,000 in January 2020 to 365,000 in December 2020 (Long 2021). Additionally, at least 5300 farmers—about 7% of Oklahoma’s farmers—have been issued the medical cannabis cultivation license (Oklahoma Medical Marijuana Authority (OMMA) 2020). Moreover, the Oklahoma medical cannabis market’s total sales reached 345 million dollars in 2019, which exceeds the total sales of milk production (209 million dollars in 2019) (USDA 2019) in the first year since legalization. These numbers imply that the cannabis demand and the corresponding supply in Oklahoma are rapidly increasing over time, yet cannabis usage is limited to medicinal use. Thus, it is evident that cannabis cultivation and sales are viable business activities in Oklahoma, notwithstanding the demand and supply restrictions by state regulation.

Recall that cannabis production and consumption in Oklahoma are restricted to medicinal use. This restriction aims to limit minors’ accessibility, hinder illegal cannabis commerce, and prevent cannabis abuse. However, the enacted OSQ denotes lenient regulations compared to other US states that legalized the use of medical cannabis. First, there is no specific requirement to issue a prescription for medicinal cannabis purchase in Oklahoma. In general, the patient needs to qualify that they have serious medical problems (e.g., cancer, HIV, rheumatoid arthritis) to purchase cannabis in the USA (e.g., California State Proposition 215, 1996; Arizona State Proposition 203, 2010). Conversely, in Oklahoma, one may not need to qualify to have a medical problem to be issued with a medical cannabis prescription. Thus, whether or not to issue a prescription for medical cannabis hinges on the subjective judgment of the local physician (OMMA 2020).

Furthermore, the OSQ 788 states that if one has been issued a medical approval once, “Renewal will be granted with resubmission of a new application. No additional criteria will be required.” Thus, there is no serious penalty for violating the criterion. The only available penalty for consumers is, at most, $400 fine for violating a possession quota. There is no permit postponement, revocation, or statement of a criminal record. These regulations are quite lenient in comparison to other medical-only states. For instance, in Florida, any individual can face up to 1 year in jail and up to $1000 fine for an arrest of minimal cannabis amounts without permit (Howell et al. 2019). These circumstances imply that, plausibly, most adults in Oklahoma may have been able to purchase and consume medical cannabis for years since the state law was passed. Even the demand for cannabis has relatively increased. For example, OMMA reported that cannabis cumulative sales tax revenue increased from $70,769 to $4,648,134 between January and June 2019 (OMMA 2021). This explains the rapidly increasing medical cannabis demand in Oklahoma by legalization.

For suppliers, the OSQ 788 states a relatively generous regulation as well. According to the OSQ 788, any individuals or entities must indicate Oklahoma residency^2^ and registration and without criminal record (no felony conviction in 5 years). In addition, cannabis suppliers (growers, and processors) in Oklahoma must pay annual fees ($2,500) and report every detail of sales and production to the state government in order for them to apply for and maintain the retailing or cultivation license. If a supplier violated any of these requirements, they would be penalized with a minimum $5,000 fine and license revocation.

Cumulatively, it is possible that the OSQ 788 can hamper illegal intervention and corresponding activities in the cannabis market but not actually control citizens’ cannabis demand. Nonetheless, supply is constrained to residency and the term of medical usage.^3^ It implies that the OSQ 788 would more likely work as an additional cost to producers and retailers (e.g., cultivating, retail, transport license fees, transport cost). Thus, the OSQ 788 could likely decrease local supply but increase the price level, which would as well shrink the market size. On average, medical-only cannabis state cities indicate higher cannabis price and fewer suppliers than legal state cities (Davenport 2019). Accordingly, we can conjecture that legalizing recreational purpose cannabis would affect the supply and demand of legalized regions and further the other states’ cannabis market as well (Hansen et al. 2020b). However, to the best of our knowledge, there are only a few studies on how the legalization act would change market quantity and profit, which is an important research gap moving forward.

Aside from medical or social controversies, recreational cannabis legalization would harm local producers and retailers by decreasing prices in the short-run (Hunt and Pacula 2017). For instance, Colorado and Washington States’ cannabis producers experienced decreased price levels since respective state government legalized recreational cannabis in 2014. Thus, there is a need to predict how the cannabis market quantity and corresponding profits would change considering Oklahoma’s legal status, allowing us to predict the change in producer surplus brought about by the legalization of recreational cannabis. Therefore, as stated before, this study estimates the retail market quantity and growers’ profit change due to the recreational cannabis legalization by simulating the legal cannabis market conditions. To make it easier to regulate the use of potentially addictive goods such as cannabis, studies that focus on subsequent welfare, producer behavior (e.g., profit, quantity change) and consumer behavior (e.g., measure willingness to pay) are required. Therefore, this study delivers a simulation analysis to reduce epistemic uncertainty from the nascent cannabis data. Its findings will be useful and provide an important springboard for further cannabis policy literature by providing producers’ perspective on cannabis legalization in the USA and other countries preparing for similar policies.

## Methodology

### Market quantity

To measure the market quantity and its change, we consider the nested constant elasticity of substitution (CES) utility function of US cannabis consumers that purchase cannabis from retail markets. We assume that when cannabis is fully legalized at federal level, there would be an interstate mailing service platform for cannabis products. This assumption is based on the cannabis market in states where cannabis is legal; the cannabis delivery service is a legally ongoing business in several states such as California (Gill and Young 2019). If products from other states are available, cannabis consumers who strongly tend to use new products (McCann and Adams 2021) would purchase products from other states online. We also take into consideration that legitimate US cannabis consumers have limited information and experience about products in other states under the current US cannabis regulation. Thus, under the federal cannabis legalization, cannabis consumers would focus on the origin of cannabis products, at least in the early stage of federal level of legalization.

The purpose and usage of medical and recreational cannabis are different, and corresponding markets are legally separated (Mead 2017). Nonetheless, in the perspective of consumers, this market difference is not as clear as with wholesalers or retailers. Lin et al. (2016) show that there is no significant difference between medical and recreational cannabis users in terms of demographic and medical characteristics. Moreover, the average content of tetrahydrocannabinol (i.e., a psychoactive compound in cannabis that produces high sensation) in medical and recreational cannabis are similar especially for the products from the online market (Cash et al. 2020), and a considerable amount of medical cannabis users use cannabis for recreational purpose (Morean and Lederman 2019; Furler et al. 2004).

Overall, our take-home message from these studies is that while the US cannabis consumers recognize medical and recreational cannabis to be different, a significant number of them may use both cannabis types for recreation. Moreover, it is plausible that such a tendency would intensify in a situation that cannabis is legalized at federal level. Given this scenario and for brevity, we employ the following assumptions in our study. First, we assume no significant quality differences (in terms of usage) between medical and recreational cannabis by the US cannabis consumers. This is plausible because unlike most recreational drugs, cannabis has a large variety of psychoactive molecules, not just one—which complicates efforts to define a dose or the cannabis equivalence of a “standard drink” (Caulkins et al. 2016). Second, we assume that cannabis consumers differentiate the medical and recreational market for a purchase decision, which is guided by legalization in a given state. Third, cannabis consumers would consider purchasing other states’ medical or recreational cannabis when it is available. By the aforementioned assumptions, a nested CES utility function proposed below is a credible framework to examine cannabis market quantity and its change due to a policy change (Sancho 2009).

**Fig. 1:**
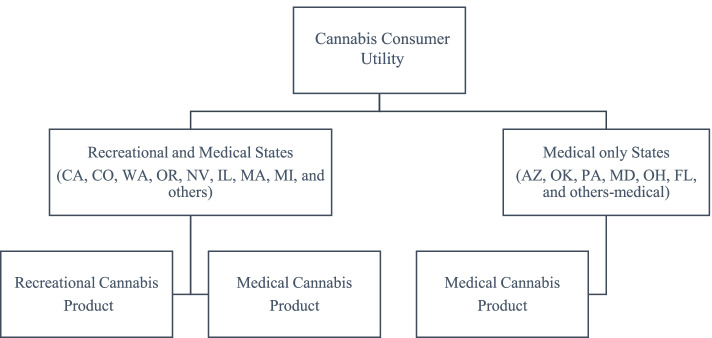
Structure of nested CES utility function of cannabis consumers

The utility maximization problem for a cannabis consumer is as follows:

1 $$\begin{array}{c}\underset Q{max}\;U=\left[\sum_{RS=1}^9\limits\left(\theta_{RS}\ast\left(\sum_{C=1}^2\limits\beta_{C,RS}\ast Q_{C,RS}^{-\rho^{RS}}\right)^\frac{-\rho^{Total}}{-\rho^{RS}}\right)+\sum_{MS=1}^7\limits\left(\theta_{MS}\ast Q_{MS}^{-\rho^{Total}}\right)\right]^{-\frac1{\rho^{Total}}},\\Subject\;to:\sum_{RS=1}^9\limits\left(\sum_{C=1}^2\limits\ P_{C,RS}\left(1+t_{C,RS}\right)\ast Q_{C,RS}\right)+\sum_{MS=1}^7\limits P_{MS}\left(1+t_{MS}\right)\ast Q_{MS}=I,\end{array}$$

where *RS* denotes states that legalized both recreational and medical cannabis (i.e., California (CA), Colorado (CO), Washington (WA), Oregon (OR), Nevada (NV), Illinois (IL), Massachusetts (MA), Michigan (MI), and Others^4^), *C* denotes the category of cannabis market (recreational and medical) within each *RS* state*, MS* denotes the medical cannabis only states (i.e., Arizona (AZ)^5^, Florida (FL), Maryland (MD), Ohio (OH), Oklahoma (OK), Pennsylvania (PA), and Others-medical^6^), *Total* denotes the whole US cannabis market, *ρ* is CES with respect to each subscript, *θ* is between-state share parameter ($\sum_{RS}\theta +\sum_{MS}\theta =1\Big)$, *β* is within-state share parameter by each market category of *RS* states ($\sum_C\beta =1\Big)$, *Q* is benchmark quantity, which is a quantity ratio by each category to total quantity, *t* is cannabis tax rate, *P* is cannabis price index, and *I* is budget. The structure of the nest follows Fig. 1.

Table 1 shows the quantity *Q* and the corresponding tax rate *t* used in Eq. (1) for each state. The tax parameter *t* reflects each state’s cannabis tax rate, taking into account each state’s cannabis laws. For example, California’s tax rate for recreational cannabis market is calculated by accounting for cannabis excise tax (15%), state retail sales tax (7.25%), local sales tax (up to 1%), and local business tax (up to 15%) (SCI Consulting Group 2017; California Department of Tax and Fee Administration 2021; Downs and Williams 2021). Therefore, a cannabis consumer would pay up to 38% in tax for California recreational cannabis products. The parameter *t* for ‘others’ and ‘others-medical’ is calculated by averaging the state tax rate of each category.

**Table 1:** 2020 medical and recreational cannabis retail market quantity and tax rate of each states

State	Legality	Quantity^a^ (%)	Tax rate (%)
California	Recreational	21.3	38.0
	Medical	4.0	15.0
Colorado	Recreational	8.2	15.0
	Medical	2.1	2.9
Washington	Recreational	6.2	37.0
	Medical	1.0	0.0
Oregon	Recreational	4.4	20.0
	Medical	0.5	0.0
Nevada	Recreational	3.4	15.0
	Medical	0.2	4.6
Illinoi	Recreational	3.1	26.0^b^
	Medical	1.7	1.0
Massachusetts	Recreational	3.3	20.0
	Medical	1.5	0.0
Michigan	Recreational	2.4	16.0
	Medical	2.2	6.0
Others	Recreational	4.9	2.8
	Medical	2.5	1.5
Arizona	Medical	5.3	9.6
Oklahoma	Medical	3.7	12.0
Pennsylvania	Medical	5.2	0.0
Maryland	Medical	2.1	0.0
Ohio	Medical	1.0	7.5
Florida	Medical	5.5	6.0
Others-medical	Medical	4.3	2.5

Source: https://equio.newfrontierdata.com/ (Frontier Financial Group 2021)

^a^Sales ratio to the total domestic market sales in 2020

^b^Based on the average of all item tax rates

We employ the standardized cannabis budget and prices, which means that the values of *P*_*C*, *RS*_, *P*_*MS*_, and *I* in the above framework are equivalently equal to one. Therefore, the calibrated optimal demand quantities via Eq. (1) would be equivalent to the optimal quantities that maximize consumers’ utility under the given assumptions.^7^ This setting allows us to present quantity changes due to policy change by state and market category (legality), which provides a clear policy outcome in terms of the market quantity. The price difference by states and legality is reflected by the difference in tax rate according to product legality by each state. Also, the online delivery assumption imposes the uniform transport cost to all consumers, which implies that a transport cost does not affect the consumer’s purchase decision in this framework. Therefore, the calibrated optimal quantity index Q^*^ would show the simulated retail market quantity due to a policy change (e.g., Oklahoma state allowing recreational cannabis) when interstate mailing service is allowed at federal level under the 2020 cannabis taxation rate of each state. Indeed, the interstate cannabis transportation is not authorized yet in the USA. However, we consider that the US federal government introduced the Marijuana Opportunity Reinvestment and Expungement (MORE) Act in 2019 that decriminalizes and de-schedules cannabis from the Controlled Substances Act. Therefore, it is plausible to conjecture that the legal or medical-only states would authorize interstate transportation in the near future. In light of this, under the CES formulation, simulating the changes in between-state elasticity of substitution would show the impact of interstate transport deregulation on cannabis markets in states if interstate cannabis transportation is authorized, which remains a judicious possibility in future.

**Fig. 2:**
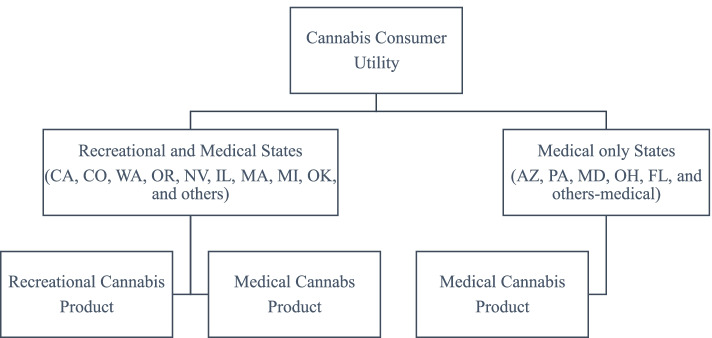
Structure of nested CES utility function of cannabis consumers after recreational cannabis is legalized in Oklahoma

We therefore apply three simulations. First, we impose a recreational cannabis market in Oklahoma to simulate the case that recreational cannabis is legalized in a medical-only state. By the first simulation, the structure of the nested CES utility function would follow Fig. 2. For this simulation, we impose an initial recreational cannabis market quantity that is twice the size of the medical cannabis market. This assumption takes into account the rapid growth of recreational cannabis markets in other legal states such as Colorado (Colorado Department of Revenue 2021). Second, we apply the heavy tax (triple of the tax rate of Oklahoma medical cannabis) on recreational cannabis market in Oklahoma. This setting is based on the medical and recreational cannabis tax rate used by US states that recently legalized recreational cannabis such as Illinois (Illinois Department of Revenue 2020). Third, we apply a relatively higher elasticity of substitution for within-state (*ρ*^*OK*^ = 2 in Eq. (1)) under the second simulation conditions to examine the case that consumers treat medical and recreational cannabis as substitutes. All the other elasticities of substitution are setup as 1.01, which imposes an imperfect substitution relationship (Armington 1969; Sancho 2009; Tohamy and Mixon Jr. 2004).

The first simulation shows the effect on the overall cannabis market if certain states legalize recreational cannabis. The second simulation indicates the outcome if the state government legalizes recreational cannabis and imposes a heavy tax on recreational cannabis just as other legal states. Finally, the third simulation shows the outcome of legalization and heavy taxation when on average, consumers perceive medical and recreational cannabis as substitutes. Each simulation is repeated under different levels of the between-state elasticity of substitution to generate a policy outcome under different degrees of interstate transport deregulation. For instance, the simulations when *ρ*^*Total*^ is 1.01 would show an Oklahoma deregulation outcome when interstate transportation is implausible, which can be represented as relatively weak substitution relationship between states’ products for cannabis consumers. On the other hand, the simulation when *ρ*^*Total*^ is 3^8^ would show the deregulation outcome when interstate cannabis transportation is flexible for consumers, and thus, a significant substitution relationship between states. Overall, these simulations illustrate the effects of legalization of recreational cannabis in within-state and between-state levels as well as how the legalization impacts change as transport regulations are eventually relaxed.

### Profit estimation

In general, indoor cannabis farming employs labor, electricity (for lighting), and other non-labor inputs such as soil, water, and nutrients (Caulkins 2010). We assume that cannabis growers use the Cobb-Douglas production technology for its simplicity. Following Chand and Kaul (1986) and for computational convenience (to reduce the number of parameters), all input prices and profit in this study are normalized by output price, i.e., divided by output price. The profit maximization problem assuming a Cobb-Douglas profit function of a cannabis grower is as follows:

2 $${\displaystyle \begin{array}{c}\underset{z}{\max }{\pi}^{\ast }={\beta}_0\prod_{j=1}^J{p_j^{\ast}}^{\beta_j}\prod_{l=1}^L{z}_l^{\gamma_l}\\ {} subject\ to:\sum_{j=1}^J\limits{\beta}_j=1,\end{array}}$$

where *π*^∗^ is normalized profit, *β*_0_ is a technical efficiency parameter, ${p}_j^{\ast }$ is a normalized *j*th input price, *β*_*j*_ is a *j*th input’s (restricted) elasticity parameter, *z*_*l*_ is endowment of a fixed factor, and *γ*_*l*_ is a fixed factor’s elasticity parameter. Capital and land are considered fixed factors in the profit function, which implies that the simulation results will be interpreted as short-run effects.

To derive the optimal input, output, and profit levels, we need price levels, efficiency, and elasticity parameters. Commonly, efficiency and elasticity parameters are estimated by regression analysis or are taken as already estimated parameters from existing literature. Alas, we could not find the Cobb-Douglas profit function study for cannabis or any other crop with similar nature (high price and addictive, such as tobacco). Therefore, we apply the efficiency parameter *β*_0_ in Nandy et al. (2021)’s meta-analysis of agricultural technical efficiency and elasticity parameters *β*_*j*_ in Schumacher and Marsh (2003)’s floriculture industry study as substitutes. For example, electricity is relatively price elastic compared to other inputs (note that the elasticity parameter indicates cross-price elasticity between inputs) while essential inputs such as labor are price inelastic. We refer to the estimated cost values from Hawken and Prieger (2013) for the input price level.^9^ The parameters and price levels are shown in Table 2. Employing the parameters from 2003 and 2013 studies may seem inadequate to replicate modern US cannabis cultivation conditions. However, considering that the technological innovation of cannabis farming has been hindered by government regulation in general (Aghion et al. 2021), we suggest that the rate of technological advances in cannabis cultivation, which is not fully legal under federal or most state laws, is relatively slower than that of crops that are legalized. Therefore, our framework would be a sufficient proxy of the average US cannabis cultivation and its outcome under the corresponding policy effects.

**Table 2:** Output price, input prices, and corresponding elasticity parameters of cannabis farming

	Price^a^ (dollars)	Input elasticity of substitution parameter^e^
Output (oz)	121.250^b^	N/A
Labor (per sf)	30.362^c^	− 0.245
Electricity (per sf)	19.035	− 1.963
Other inputs^d^ (per sf)	18.554	− 1.444

Input prices are based on the one year production level of the 1500 sf indoor facility (Hawken and Prieger 2013)

^a^Input prices are based on the cost per square foot

^b^The average 2020 cannabis wholesale market price per ounce (source: Branfalt (2020) and Leaflink Insights (2020))

^c^Includes general agricultural work, trimming, and management labor cost

^d^The average cost of water, soil, CO_2_, nutrients, pesticide, and rent

^e^Source: Schumacher and Marsh (2003)

We apply four scenarios to simulate the legalization conditions for each cannabis grower. First, we apply a per-ounce cultivation tax (9.65 US dollars per ounce) on recreational cannabis growers. This is to replicate the existing legal state cannabis policy, such as the case of California legalization in 2016 (California Department of Tax and Fee Administration 2021). Second, we employ a 10% increase in cannabis price, which implies a price bubble in the early stage of a new market, to simulate an initial state of recreational cannabis legalization (Doraszelski et al. 2018; Hansen et al. 2020a). Third, we apply a 10% decrease in cannabis price to simulate the case when the market price is stabilized after recreational cannabis legalization^10^ (Shover and Humphreys 2019) for growers. Fourth, we apply the reduced input-price case, to simulate a decreased cost due to technological development through legalization (Caulkins 2010; Kagia et al. 2020). This framework is expected to have two outcomes. First, the approach will illustrate specifically how much a cultivation tax would affect new cannabis growers when recreational cannabis is legalized in a specific region or country. Second, the input-price reduction scenario will demonstrate how much of a cost reduction is required for new recreational cannabis farms to ensure a profit level similar to the existing medical cannabis farming. Therefore, using the estimated average measures for US cannabis cultivation in Table 2, simulation results will reflect the effect of the legalization on average US cannabis farms. This information is essential to the local authorities to set a policy that could guarantee tax revenues while minimizing the exit rate of producers in the legalized cannabis market (Kilmer 2014). As for software, all simulations were executed in the Generalized Algebraic Modeling Systems (GAMS) program using a non-linear programming procedure. More details about GAMS can be found in McCarl and Spreen (1997).

## Results and discussion

We present cannabis market quantity changes due to legalization under different potential interstate transport deregulation scenarios in Tables 3 and 4. Each table reports the calibrated optimal market quantity index Q^*^^11^, between-state quantity index parameter *θ*, and within-state quantity index parameter *β*.^12^ Table 3 shows the legalization and corresponding taxation outcome when interstate transport is implausible (*ρ*^*Total*^ = 1.01). Sub-scenario 1-2 in Table 3 shows that if recreational cannabis is legalized in Oklahoma—a medical-only state—it would decrease the existing medical cannabis market quantity index ${Q}_{Medical, OK}^{\ast }$ from 0.033 to 0.030, which is a 9.1% decrease. Also, such legalization in Oklahoma would significantly decrease other states’ market quantity. For instance, California recreational cannabis market quantity would decrease from 0.154 to 0.143, which is a 7.1% decrease.^13^

**Table 3:** Indexed cannabis retail market quantities by state, market, and scenario: under weak substitution (*ρ*^*Total*^ = 1.01)

State	Legality	Scenario 1-1: Base model			Scenario 1-2: Legalize recreational cannabis in Oklahoma^a^			Scenario 1-3: Legalize recreational cannabis in Oklahoma + heavy tax on recreational^b^			Scenario 1-4: Legalize recreational cannabis in Oklahoma+ heavy tax on recreational + medical and recreational cannabises are substitutes^c^		
		Q^*^	*θ*	*β*	Q^*^	*θ*	*β*	Q^*^	*θ*	*β*	Q^*^	*θ*	*β*
California	Recreational	0.154	0.251	0.839	0.143	0.234	0.839	0.143	0.234	0.839	0.143	0.234	0.839
	Medical	0.034		0.160	0.032		0.160	0.032		0.160	0.032		0.160
Colorado	Recreational	0.071	0.103	0.794	0.066	0.096	0.794	0.066	0.096	0.794	0.066	0.096	0.794
	Medical	0.020		0.206	0.019		0.206	0.019		0.206	0.019		0.206
Washington	Recreational	0.045	0.072	0.858	0.042	0.068	0.858	0.042	0.068	0.858	0.042	0.068	0.858
	Medical	0.010		0.141	0.009		0.141	0.009		0.141	0.009		0.141
Oregon	Recreational	0.036	0.049	0.895	0.034	0.045	0.895	0.034	0.045	0.895	0.034	0.045	0.895
	Medical	0.005		0.104	0.004		0.104	0.004		0.104	0.004		0.104
Nevada	Recreational	0.029	0.036	0.942	0.027	0.033	0.942	0.027	0.033	0.942	0.027	0.033	0.942
	Medical	0.001		0.057	0.001		0.057	0.001		0.057	0.001		0.057
Illinoi	Recreational	0.016	0.048	0.355	0.015	0.045	0.355	0.015	0.045	0.355	0.015	0.045	0.355
	Medical	0.024		0.644	0.022		0.644	0.022		0.644	0.022		0.644
Massachusetts	Recreational	0.027	0.047	0.685	0.025	0.045	0.685	0.025	0.045	0.685	0.025	0.045	0.685
	Medical	0.015		0.314	0.014		0.314	0.014		0.314	0.014		0.314
Michigan	Recreational	0.020	0.046	0.512	0.019	0.043	0.512	0.019	0.043	0.512	0.019	0.043	0.512
	Medical	0.020		0.478	0.019		0.478	0.019		0.478	0.019		0.478
Others	Recreational	0.047	0.074	0.660	0.044	0.069	0.660	0.044	0.069	0.660	0.044	0.069	0.660
	Medical	0.024		0.339	0.023		0.339	0.023		0.339	0.023		0.339
Oklahoma	Recreational	–	0.038	–	0.061	0.104	0.665	0.050	0.103	0.665	0.047	0.103	0.585
	Medical	0.033		–	0.030		0.334	0.030		0.334	0.034		0.414
Arizona	Medical	0.048	0.054	–	0.045	0.049	–	0.045	0.049	–	0.045	0.049	-
Pennsylvania	Medical	0.052	0.052	–	0.048	0.048	–	0.048	0.048	–	0.048	0.048	–
Maryland	Medical	0.021	0.021	–	0.019	0.019	–	0.019	0.019	–	0.019	0.019	–
Ohio	Medical	0.009	0.01	–	0.008	0.009	–	0.008	0.009	–	0.008	0.009	–
Florida	Medical	0.051	0.055	–	0.048	0.052	–	0.048	0.052	–	0.048	0.052	–
Others-medical	Medical	0.042	0.043	–	0.039	0.040	–	0.039	0.040	–	0.039	0.040	–
Total	–	0.854	0.999	–	0.856	0.999	–	0.845	0.999	–	0.846	0.999	–

^a^Assume the double market quantity of 2020 medical cannabis in Oklahoma

^b^Assume the triple tax of 2020 medical cannabis in Oklahoma

^c^*ρ*^*OK*^ = 2

**Table 4:** Indexed cannabis retail market quantities by state, market, and scenario: under significant substitution (*ρ*^*Total*^ = 3)

State	Legality	Scenario 2-1: Base model			Scenario 2-2: Legalize recreational cannabis in Oklahoma^a^			Scenario 2-3: Legalize recreational cannabis in Oklahoma + heavy tax on recreational^b^			Scenario 2-4: Legalize recreational cannabis in Oklahoma+ heavy tax on recreational + medical and recreational cannabises are substitutes^c^		
		Q^*^	*θ*	*β*	Q^*^	*θ*	*β*	Q^*^	*θ*	*β*	Q^*^	*θ*	*β*
California	Recreational	0.112	0.114	0.839	0.104	0.107	0.839	0.107	0.107	0.839	0.106	0.107	0.839
	Medical	0.025		0.160	0.023		0.160	0.024		0.160	0.024		0.160
Colorado	Recreational	0.074	0.088	0.794	0.068	0.083	0.794	0.070	0.083	0.794	0.070	0.083	0.794
	Medical	0.021		0.206	0.020		0.206	0.020		0.206	0.020		0.206
Washington	Recreational	0.034	0.073	0.858	0.032	0.069	0.858	0.033	0.069	0.858	0.033	0.069	0.858
	Medical	0.008		0.141	0.007		0.141	0.007		0.141	0.007		0.141
Oregon	Recreational	0.034	0.061	0.895	0.032	0.058	0.895	0.033	0.058	0.895	0.033	0.058	0.895
	Medical	0.005		0.104	0.004		0.104	0.004		0.104	0.004		0.104
Nevada	Recreational	0.029	0.050	0.942	0.027	0.048	0.942	0.028	0.048	0.942	0.028	0.048	0.942
	Medical	0.002		0.057	0.002		0.057	0.002		0.057	0.002		0.057
Illinoi	Recreational	0.016	0.074	0.355	0.015	0.071	0.355	0.015	0.071	0.355	0.015	0.071	0.355
	Medical	0.024		0.644	0.022		0.644	0.022		0.644	0.022		0.644
Massachusetts	Recreational	0.028	0.072	0.685	0.026	0.069	0.685	0.027	0.069	0.685	0.027	0.069	0.685
	Medical	0.015		0.314	0.014		0.314	0.015		0.314	0.014		0.314
Michigan	Recreational	0.022	0.075	0.512	0.020	0.072	0.512	0.021	0.072	0.512	0.021	0.072	0.512
	Medical	0.022		0.478	0.020		0.478	0.021		0.478	0.021		0.478
Others	Recreational	0.059	0.085	0.660	0.055	0.080	0.660	0.056	0.080	0.660	0.056	0.080	0.660
	Medical	0.031		0.339	0.028		0.339	0.029		0.339	0.029		0.339
Oklahoma	Recreational	–	0.044	–	0.064	0.092	0.665	0.042	0.092	0.665	0.039	0.092	0.585
	Medical	0.034		–	0.032		0.334	0.025		0.334	0.029		0.414
Arizona	Medical	0.052	0.049	–	0.049	0.047	–	0.050	0.047	–	0.050	0.047	–
Pennsylvania	Medical	0.068	0.049	–	0.063	0.046	–	0.065	0.046	–	0.064	0.046	–
Maryland	Medical	0.027	0.036	–	0.025	0.034	–	0.026	0.034	–	0.026	0.034	–
Ohio	Medical	0.010	0.028	–	0.010	0.027	–	0.010	0.027	–	0.010	0.027	–
Florida	Medical	0.060	0.050	–	0.056	0.047	–	0.057	0.047	–	0.057	0.047	–
Others-medical	Medical	0.070	0.051	–	0.065	0.048	–	0.067	0.048	–	0.067	0.048	–
Total	–	0.882	0.999	–	0.883	0.998	–	0.876	0.998	–	0.874	0.998	–

^a^Assume the double quantity of 2020 medical cannabis in Oklahoma

^b^Assume the triple tax of 2020 medical cannabis in Oklahoma

^c^*ρ*^*OK*^ = 2

**Fig. 3:**
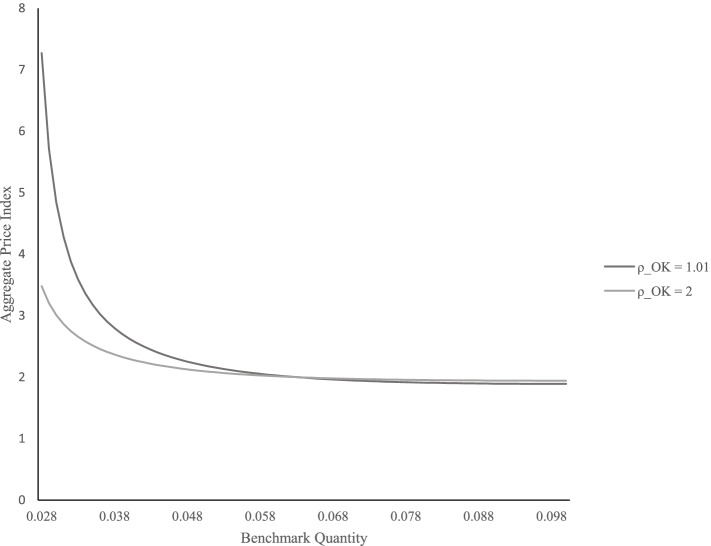
Demand curve shift of Oklahoma cannabis market: due to the differences in elasticity of substitution between medical and recreational cannabis

Sub-scenario 1-3 in Table 3 further shows that if the Oklahoma government charged a heavy excise tax on recreational cannabis, it would decrease the recreational cannabis market quantity in the state yet, the state would not recover its medical cannabis market quantity. Nonetheless, if Oklahoma cannabis consumers consider medical and recreational cannabis as substitutes (i.e., sub-scenario 1-4 in Table 3), a heavy taxation on recreational cannabis would induce consumers to purchase less recreational cannabis (i.e., ${Q}_{Recreational, OK}^{\ast }$ has changed from 0.050 to 0.047) and more medical cannabis (i.e., ${Q}_{Medical, OK}^{\ast }$ has changed from 0.030 to 0.034) than the case of sub-scenario 1-3. Figure 3 shows how the local consumers’ substitution behavior affects the taxation policy outcome.

Figure 3 shows a shift in the simulated Oklahoma cannabis market demand curve due to Oklahoma cannabis consumers’ perspective on medical and recreational cannabis products.^14^^,^^15^ Notice that under the assumption of weak substitution between medical and recreational cannabis (*ρ*^*OK*^ = 1.01), the demand curve’s slope is steeper, i.e., lower demand price elasticity than a relatively significant substitution (*ρ*^*OK*^ = 2) condition. In addition, we observe that when the quantity exceeds a certain level, all the demand curves show a stronger price elasticity of demand regardless of the difference in degree of substitution. Therefore, we conclude that local cannabis consumers would be sensitive to taxation policies if medical cannabis and recreational cannabis are treated as significant substitutes or when a supply is above a certain level. Overall, legalizing recreational cannabis with a heavy excise tax may harm the existing medical cannabis market in Oklahoma. Nonetheless, it would benefit the existing medical cannabis market when local consumers consider medical and recreational cannabis as significant substitutes.

For other states, sub-scenario 1-3 and 1-4 would barely affect the other states’ market quantities (smaller than 0.001) on average. Our findings suggest that when interstate transportation is available yet implausible, the local (in a newly legalized state) taxation policy and local consumer’s substitution behavior would mainly affect the local cannabis market quantity. In other words, the spillover effect of local cannabis policy to other states would be negligible.

We also observe an interesting change in the total quantity index (last row of Table 3) through from sub-scenario 1-1 to 1-4. The initial total quantity is 0.854, which is smaller than 1, and may imply a market-sales loss due to taxation for each state (Mace et al. 2020). By legalization (sub-scenario 1-2), the total market quantity has increased from 0.854 to 0.856 (0.2% increase)—the overall market quantity does not increase as much as the quantity of the newly legalized market due to imperfect substitution. In addition, we observe that heavy taxation (sub-scenario 1-3) even regresses the total market quantity to the before-legalization level: from 0.856 to 0.845 (1.2% decrease). Besides, such market quantity decrease from heavy taxation is irrelevant to the local consumers’ substitution behavior (sub-scenario 1-4). These results imply that when interstate transportation is implausible, the increase in total demand due to the new market is insignificant and the decrease in the total demand due to taxation could be large enough to regress to the before-legalization demand level.

Table 4 shows the potential outcome from legalization and the corresponding taxation when interstate cannabis transportation is flexible (*ρ*^*Total*^ = 3). From sub-scenario 2-1 to 2-2, the implication for Oklahoma is quite similar to the findings in Table 3. That is, when interstate transportation is available and a particular medical-only state legalizes a recreational cannabis market, a significant amount of other states’ cannabis consumers purchase the products from a newly legalized market. The notable difference is that the cannabis market quantities under the heavy tax rates (California and Washington) would decline significantly, while the quantities of all other states would increase or remain nearly the same compared to the case of sub-scenario 1-1. This implies that when interstate transportation is flexible, the cannabis consumers in high-tax states would quickly substitute with the other states’ product to avoid heavy taxation. On the other hand, we observe that the negative spillover effect by legalization remains almost the same as previous sub-scenario cases. For instance, in sub-scenario 2-1, the California recreational cannabis market quantity is 0.112 units, noticeably a smaller quantity than in sub-scenario 1-1. However, the quantity decrease from 0.112 to 0.104, a 7.2% decrease by legalization (sub-scenario 2-2), which is similar to the difference in sub-scenario 1-1 and 1-2 outcomes.

In addition, we observe that the excise tax effect (sub-scenario 2-3) on Oklahoma’s recreational cannabis market quantity would decrease from 0.064 to 0.042, while the medical cannabis market quantity would decrease from 0.032 to 0.025. This suggests that local recreational and medical cannabis consumers would be more inclined to substitute products from other states when both interstate shipping is flexible and if newly legalized states impose heavy taxes.

Aside from a newly legalized state, we can also observe a significant response from the other US states’ markets: a case whereby recreational cannabis is legalized, and corresponding local taxation policy is executed. For example, in sub-scenario 2-3, we find that the California’s recreational cannabis market quantity would increase from 0.104 to 0.107 (3% increase) as a result of a heavy tax on a new recreational cannabis market in Oklahoma, which is a significant substitution behavior compared to the case in Table 3. On the other hand, the substitution behavior of Oklahoma consumers (i.e., sub-scenario 2-4) would have no meaningful effects on other states just as sub-scenario 1-4. This implies that when interstate transportation is flexible and a heavy tax is imposed, Oklahoma recreational cannabis consumers would more likely substitute local cannabis products for other states’ cannabis products. Indeed, the local taxation policy effect on the other states is at most 3%, which seems negligible. However the elasticity of substitution value of 3 (*ρ*^*Total*^ = 3) represents the average level of substitutability in US interstate trade products (Yilmazkuday 2012). Therefore, if a cannabis product is more substitutable than the average level of interstate products, the spillover effect of local policy on other states could be considerably significant.

In terms of the total market quantity, we observe a larger initial total market across all sub-scenario in Table 4. For instance, sub-scenario 2-1 shows a total quantity 0.882, which is larger than in sub-scenario 1-1. This shows that when interstate transportation is flexible, the total market quantity loss by spillover effect due to policy (e.g., heavy taxation) would be compensated with significant substitution between states, as heavy tax states’ consumers (e.g., California) would become more flexible to substitute for other state products.

Table 5 shows the profit-maximizing solution of cannabis growers using a Cobb-Douglas production technology according to each cannabis policy simulation. The base model (scenario 1) results indicate that when cannabis growers have no cannabis growing restraining policies, they would realize cannabis yield of 5690 ounces for a 1500 square-foot (sf) facility (0.23 pound per sf), *ceteris paribus*. This yield level is higher than Caulkins’ (2010) estimates (0.10 pound per sf) and Wilson et al.’s (2019) survey results (0.16 pound per sf) for an indoor facility case. Our finding implies that currently operating cannabis farms may not realize such an optimal level of production due to external factors such as regulations. Additional scenarios in Table 5 clearly support this finding. Scenario 2 indicates a per-ounce tax reduces profit and yield by 27% yet such taxation would provide a huge tax revenue ($39,847 per cannabis farm) for the local government. On the other hand, the early legalization stage with a higher price (10% increased output price in scenario 3) shows considerably increased profit, yield, and tax revenue. This suggests that at the early stage of legalization, taxation policy would not be a big issue in terms of producers and local government’s tax revenue. Nonetheless, the subsequent legalization case after the market price is stabilized (i.e., 10% price decrease) shows a huge negative impact on profit, yield and tax revenue.

**Table 5:** Simulated per-grower’s accounting outcomes and corresponding tax revenue

Variable	Scenario 1: Base model	Scenario 2: Per ounce taxation^a^		Scenario 3: Per ounce taxation + 10% increased output price		Scenario 4: Per ounce taxation + 10% decreased output price		Scenario 5-1: Per ounce taxation +10% decreased output price +5% decreased input price		Scenario 5-2: Per ounce taxation +10% decreased output price +10% decreased input price	
Profit (USD)	145252.58	105405.49	(− 27%)	168418.67	(16%)	62655.52	(− 57%)	75563.62	(− 48%)	92058.88	(− 37%)
Output level (oz)	5690.23	4129.23	(− 27%)	5597.96	(− 2%)	2727.24	(− 52%)	3289.09	(− 42%)	4007.09	(− 30%)
Total variable cost (USD)	530462.41	384940.84	(− 27%)	615064.98	(16%)	228817.97	(− 57%)	275958.33	(− 48%)	336199.05	(− 37%)
Tax revenue (USD)	−	39847.09	−	57880.32	(45%)	26317.84	(− 34%)	31739.76	(− 20%)	38668.44	(− 3%)

Numbers in parenthesis indicate the percentage change from scenario 1 (for the tax revenue part, the percentage change from scenario 2)

^a^9.65 US dollars per ounce

Pacula (2010) argues that a 10% price decrease would lead to a 3–5% increase in the number of cannabis consumers. However, scenario 4 results in Table 5 show that legalization after cannabis price is stabilized would lead to a 52%, 57%, and 34% reduction in yield, profits, and tax revenue, respectively—a loss that would be too large to compensate for a 5% increase in consumers. These results suggest that if the State government plans to legalize recreational cannabis and implement a taxation policy accordingly, some level of technological advancement or government support is needed to prevent cannabis producers from leaving the market or even moving to illicit markets (Kilmer 2014; Bodwitch et al. 2019). Scenarios 5-1 and 5-2 in Table 5 provide a hint for this problem. Scenarios 5-1 and 5-2 show that input prices would decrease by 5% and 10%, respectively, due to legalization-induced technological innovation or local government support. The 5% input price reduction would subsequently recover 10% of profit, yield, and tax revenue as shown in scenario 4 on average. Moreover, a 10% cost reduction shows a recovery to scenario 2 levels of profit, yield, and tax revenue.^16^ Overall, results from scenario 5 indicate that if there is some cost-saving innovation through the legalization process (at least 10% of input price reduction in this case), profit losses from taxation and price increases caused by recreational cannabis legalization may be compensated.

## Conclusion and policy implications

Cannabis legalization is one of the controversial subjects as there is no clear evidence of positive or negative consequences. One of the main reasons is that while cannabis legalization almost certainly implies legal cannabis production, changes in the cannabis market supply-side are often neglected. Therefore, with scanty evidence of legalization related supply-side effects, obtaining a clear vision of a cannabis legalization policy and its consequences may be elusive. This study contributes to the scanty literature by being the first to simulate the cannabis market’s supply-side effects from legalization. This study simulates the legalization of recreational cannabis so as to provide an overview of potential changes in the retail market quantity, growers’ profits, and the corresponding government tax revenue from interstate transport deregulation. We applied the significant tax rate on a newly legalized recreational cannabis market to simulate the legalization condition.

The following conclusions can be drawn from this study. First, if a medical-only state goes on to legalize recreational cannabis, legalization would lead to negative spillover effects on both the existing medical cannabis market in a legalized region and the cannabis markets in other states. The legalization of recreational cannabis would generally decrease most of a state’s cannabis market quantity, especially for those states with large and heavily taxed markets such as California. Second, an excise taxation policy in a legalized recreational market may induce consumers to substitute local recreational cannabis with other states’ products, yet the degree of the between-state substitution effect would be negligible when the between-state products are weakly substitutable (i.e., implausible interstate transportation) for consumers. If a newly legalized state’s consumers consider medical and recreational cannabis as significant substitutes—which people tend to do without a doctor’s recommendation (Lloyd et al. 2020), taxation policy would mainly penalize the newly legalized recreational cannabis markets. The demand curve estimation with aggregate price index and benchmark quantity shows that a lower elasticity of substitution between medical and recreational cannabis stimulates a lower demand price elasticity in a cannabis market. Third, the spillover effects of cannabis legalization vary by the level of deregulation of interstate transportation. As transportation between states becomes flexible, the spillover effects of taxation to other states would become meaningful, and market quantities in potentially newly legalized states would become more sensitive to legalization and taxation policies, especially when consumers consider medical and recreational cannabis as substitutes.

In terms of profit of the cannabis growers, legalization and the corresponding taxation would rapidly decrease the existing cannabis growers’ yield and profit by more than 50% after the initial price bubble disappears, everything else held constant. After accounting for input cost reduction effect by legalization, we found that legalization would however recover a huge amount of profit for growers and tax revenue for local government. That is, while legalization could increase producer surplus through increased overall market sales in a legalized state, each farming household would be hampered by decreased profits unless technological development or government support is in place. In sum, legalizing recreational cannabis would increase the market sales in a legalized state but would likely hamper other states with a large market size. Despite that, the flexible interstate transportation condition would significantly increase the overall demand quantity. Still, it would likely harm the cannabis growers’ profit in the short-run unless followed by technological development in terms of cost reduction.

Based on these findings, a policy for legalizing recreational cannabis has potential as it could generate tax revenue to fund critical government projects and services. However, such legalization should be done carefully because heavy excise taxes would decrease the newly legalized cannabis market demand and growers’ profit, which may imply cannabis producers and consumers potentially moving to the illicit market (Hsiang and Sekar 2016). Additionally, providing more flexible interstate transportation is also encouraged as it would mitigate the total market demand loss by legalizing medical or recreational cannabis and the corresponding taxation. Thus, policymakers would have to compromise between the levels of interstate transportation and taxation to ensure that cannabis producers also realize some profit within the cannabis supply chain.

Limitations of our study are as follows. First, due to lack of empirical data, this study relied on simulations of different scenarios such as considering retail market quantity changes based on the price-taking consumers’ utility maximization. This could have contributed to some bias in the results, and thus, the interpretation of our results with caution is encouraged. Second, our study lacks the consumer-side welfare analysis whose addition would have helped grasp the potential total welfare effect of legalization. Additionally, accounting for market power with respect to producer, processor, and retailer sides would be necessary to provide a better policy outcome in the cannabis industry. Third, we could not account for the legalization effect on the illicit cannabis market, which could be a valuable implication for local governments considering legalization (Bodwitch et al. 2019). Moreover, cannabis products contain multiple psychoactive molecules with differing psychological and physiological effects (Chandra et al. 2017). Our study did not consider the effects that cannabis quality differences could have on our results. Admittedly, this is an important caveat.

Therefore, using real-world data sets, applying econometric models while considering the market power of producers and retailers and their interrelationships, and accounting for impacts of quality differences in cannabis products are interesting aspects for future studies. Regardless of these caveats, our results are the first based on a simulated cannabis market behavior, assuming market conditions in our setting—which are plausible aspects related to the cannabis industry and its legalization. In short, this study provides a plausible springboard upon which future related studies can be drawn.

## Data Availability

The code will be available upon reasonable request from the corresponding author.

## Footnotes

1 We are grateful to one anonymous reviewer for suggesting this statement.

2 The non-Oklahoma resident’s ownership of entity may not exceed 25%.

3 Medical marijuana is not able to sale for other purposes, such as recreation.

4 “Others” is the aggregated quantity of states that indicate negligible but non-zero cannabis market quantity (in between 0 and 1% of sales ratio to the total market sales) for both medical and recreational market.

5 Arizona is classified medical-only state in our framework because no actual recreational cannabis market has formed in 2020, the current study’s base year.

6 Just as “Others,” “Others-medical” is the aggregated quantity of medical-only states with insignificant market quantity.

7 Therefore, the optimal market quantity of this study is based on given (exogenous) price levels and thus price-taking cannabis consumers’ utility-maximizing optimal solution.

8 The average value of elasticities of substitution across interstate products that listed in Standard Classification of Transported Goods (SCTG) (Yilmazkuday 2012). Therefore, this simulation would show how policy change affect each state's market quantity when cannabis products have an average level of interstate substitutability in US.

9 Just as a market quantity simulation part, this profit maximization process is based on the given price levels, which impose price-taking cannabis growers. Therefore, the calibrated values of this study represents the price-taking cannabis growers’ profit-maximizing behavior, which could be considered as a partial equilibrium outcome of perfectly competitive cannabis market.

10 Considering that the cannabis price started to be stabilized after less than a year (insufficient time-lapse to consider as a long-run) in Washington legalization case (Hansen et al. 2020a), we consider this scenario as a part of short-run simulation.

11 In CES framework with standardized price level, the sum of optimal market quantity indices *Q*^***^ should be equal to one. However, by the price difference between states due to taxation, the sum of *Q*^***^ is less than one in the last rows of Tables 3 and 4. This may imply that due to taxation, each state’s market quantity does not reach the ideal level, and thus a deadweight loss. However, deadweight loss is not discussed in depth as it is beyond the objective of this study.

12 Since we employ the standardized price in Eq. (1), *θ* and *β* are only affected by elasticities of substitution and given quantity data. Therefore, the calibrated *θ* and *β* would indicate between- and within-state quantity proportions without considering the maximization of consumer utility due to the difference in tax rates between states.

13 In Hansen et al. (2020b)’s Washington legalization study case, between 8.1 and 11.5% of Washington’s cannabis tax revenue comes from the Oregon’s cannabis consumers. Considering that Washington’s population is 1.8 times larger than Oregon and California population is 10 times larger than Oklahoma, the Oklahoma legalization impact on California market quantity would be close to $11.5\%\times \left(\frac{1.8}{10}\right)=2.07\%$, or at least close to $8.1\%\times \left(\frac{1.8}{10}\right)=1.46\%$. In terms of quantity ratio to the total quantity index, the percentage change by Oklahoma legalization in California could be represented as $\left(\frac{0.154}{0.854}\right)-\left(\frac{0.143}{0.856}\right)=0.013=1.3\%$. Therefore, we consider our result as consistent with the previous literature. We sincerely appreciate one anonymous reviewer for suggesting this statement.

14 In Fig. 3, the *x*-axis is the benchmark demand quantity and the *y*-axis is a calibrated within-state aggregate price index representing the local relative price level of Oklahoma cannabis products, *ceteris paribus*.

15 This estimation of demand curve is based on the benchmark quantities and corresponding price index function (e.g., Keller 1976; Rutherford 2002). The price index for Oklahoma ${P}_{OK}^{\ast }$ is calibrated as follows:

$${P}_{OK}^{\ast }=\left[\frac{\sum_C{Q}_{C, OK}}{{\left(\sum_C{\beta}_{C, OK}\ast {\left({Q}_{C, OK}\right)}^{-{\rho}_{OK}}\right)}^{-\frac{1}{\rho_{OK}}}}\right].$$

The numerator on the right-hand side is the total available quantity while the denominator is an aggregated consumption quantity due to a given within-state elasticity of substitution. Since the total available quantity is given, the price index and consumption quantity are negatively correlated. Therefore, the price index function could be considered as a pseudo-demand function that indicates a relationship between the demand quantity and its determinants, such as within-state elasticity of substitution. Accordingly, the calibrated price index could be interpreted as local price received in Oklahoma due to local consumers’ substitution behavior.

16 Interestingly, scenario 4 shows yield quantity of 0.11 pounds per sf while scenario 5-2 shows yield level of 0.16 pounds per sf, respectively. These findings corroborate with Toonen et al.’s (2006) (relatively outmoded) illicit cannabis cultivation and Wilson et al.’s (2019) findings about modern legal cannabis cultivation yield level. These findings show that scenario 4 replicates the early stage condition of cannabis cultivation whereby there are no advanced cultivation techniques or legality. To the contrary, scenario 5 replicates modern farming environment with the latest technology for legalized cannabis cultivation.
